# Supplementary material for: Cross-sectional comparisons of sodium content in processed meat and fish products among five countries: potential for feasible targets and reformulation
Source: BMJ Open. 2021 Sep 14;11(10):e046412. doi: 10.1136/bmjopen-2020-046412 (PMC8559105; doi:10.1136/bmjopen-2020-046412)
Supplement: Author's manuscript [file bmjopen-2020-046412.draft_revisions.pdf]

BMJ Open is committed to open peer review. As part of this commitment we make the peer review history of every article we publish publicly available.

When an article is published we post the peer reviewers' comments and the authors' responses online. We also post the versions of the paper that were used during peer review. These are the versions that the peer review comments apply to.

The versions of the paper that follow are the versions that were submitted during the peer review process. They are not the versions of record or the final published versions. They should not be cited or distributed as the published version of this manuscript.

BMJ Open is an open access journal and the full, final, typeset and author-corrected version of record of the manuscript is available on our site with no access controls, subscription charges or pay-per-view fees (http://bmjopen.bmj.com).

If you have any questions on BMJ Open's open peer review process please email info.bmjopen@bmj.com

BMJ Open

Comparisons of sodium content in processed meat and fish products among five countries   potential for reformulation

Journal:	*BMJ Open*
Manuscript ID	bmjopen-2020-046412
Article Type:	Original research
Date Submitted by the Author:	31-Oct-2020
Complete List of Authors:	Song, Yuzhu; Hebei Medical University,<br>Li, Yuan; The George Institute for Global Health, Peking University Health Science Centre<br>Guo, Chunlei; The George Institute for Global Health, Peking University Health Science Centre<br>Wang, Yishan; University of Hong Kong<br>Huang, Liping; The George Institute for Global Heath, Food Policy<br>Tan, Monique; Wolfson Institute of Preventive Medicine, Barts and The London School of Medicine & Dentistry, Queen Mary University of London, Centre for Environmental and Preventive Medicine<br>He, Feng; Queen Mary University of London, Wolfson Institute of Preventive Medicine<br>Harris, Terry<br>MacGregor, Graham; Queen Mary University of London, Wolfson Institute of Preventive Medicine<br>Ding, Jingmin; The George Institute for Global Health, Peking University Health Science Centre<br>Dong, Le; The George Institute for Global Health, Peking University Health Science Centre<br>Zhang, Puhong ; The George Institute at Peking University Health Science Center, Diabetes Program<br>Ma, Yuxia; Hebei Medical University
Keywords:	EPIDEMIOLOGY, Health policy < HEALTH SERVICES ADMINISTRATION & MANAGEMENT, Public health < INFECTIOUS DISEASES, PREVENTIVE MEDICINE

SCHOLARONE™
Manuscripts

I, the Submitting Author has the right to grant and does grant on behalf of all authors of the Work (as defined in the below author licence), an exclusive licence and/or a non-exclusive licence for contributions from authors who are: i) UK Crown employees; ii) where BMJ has agreed a CC-BY licence shall apply, and/or iii) in accordance with the terms applicable for US Federal Government officers or employees acting as part of their official duties; on a worldwide, perpetual, irrevocable, royalty-free basis to BMJ Publishing Group Ltd ("BMJ") its licensees and where the relevant Journal is co-owned by BMJ to the co-owners of the Journal, to publish the Work in this journal and any other BMJ products and to exploit all rights, as set out in our licence.

The Submitting Author accepts and understands that any supply made under these terms is made by BMJ to the Submitting Author unless you are acting as an employee on behalf of your employer or a postgraduate student of an affiliated institution which is paying any applicable article publishing charge ("APC") for Open Access articles. Where the Submitting Author wishes to make the Work available on an Open Access basis (and intends to pay the relevant APC), the terms of reuse of such Open Access shall be governed by a Creative Commons licence – details of these licences and which Creative Commons licence will apply to this Work are set out in our licence referred to above.

Other than as permitted in any relevant BMJ Author's Self Archiving Policies, I confirm this Work has not been accepted for publication elsewhere, is not being considered for publication elsewhere and does not duplicate material already published. I confirm all authors consent to publication of this Work and authorise the granting of this licence.

For peer review only

Comparisons of sodium content in processed meat and fish products among five countries potential for reformulation

Corresponding authors: Puhong Zhang: The George Institute for Global Health at Peking University Health Science Center ; zpuhong@georgeinstitute.org.cn; and Yuxia Ma: Hebei Medical University; mayuxia@hebmu.edu.cn;

Co-authors:

1 Yuzhu Song: Department of nutrition and food hygiene; Hebei Medical University; Shijiazhuang; China.

2 Yuan Li : The George Institute for Global Health at Peking University Health Science Center; Beijing; China.

3 Chunlei Guo: The George Institute for Global Health at Peking University Health Science Center; Beijing; China.

4 Yishan Wang : School of Public Health, LKS Faculty of Medicine, The University of Hong Kong, Hong Kong, China.

5 Liping Huang : The George Institute for Global Health | AUSTRALIA; Australia.

6 Monique Tan : Wolfson Institute of Preventive Medicine, Barts and The London School of Medicine & Dentistry, Queen Mary University of London, London, UK.

7 Feng J He : Wolfson Institute of Preventive Medicine, Barts and The London School of Medicine & Dentistry, Queen Mary University of London, London, UK.

8 Terry Harris : Discovery Vitality, Johannesburg, South Africa.

For peer review only

9 Graham A MacGregor : Wolfson Institute of Preventive Medicine, Barts and The London School of Medicine & Dentistry, Queen Mary University of London, London, UK.

10 Jingmin Ding: The George Institute for Global Health at Peking University Health Science Center; Beijing; China.

11 Le Dong: The George Institute for Global Health at Peking University Health Science Center; Beijing; China.

12 Puhong Zhang: The George Institute for Global Health at Peking University Health Science Center; Beijing; China.

13 Yuxia Ma: Department of nutrition and food hygiene; Hebei Medical University; Shijiazhuang; China.

Word count:3268

For peer review only

ABSTRACT

Introduction: Reducing sodium intake has been identified as a highly cost-effective strategy to improve public health. This study aims to compare the sodium content in processed meat and fish products among five countries and provide a basis for feasible strategies of sodium reduction in such products.

Methods: Nutrition information of 19601 meat and 6899 fish products was collected using the FoodSwitch mobile application from China, the United Kingdom (UK), Australia, South Africa and the United States (US) from 2012 to 2018 and analyzed for cross-sectional comparisons.

Results: The results showed that processed meat and fish products combined in China had the highest sodium level (median 1050 mg/100g, interquartile range [IQR]: 774-1473), followed by the US, South Africa, Australia, with the lowest levels found in UK (432 mg/100g, IQR: 236-786) (p<0.001). Similar variations, i.e. a 2-3-fold difference of sodium content between the highest and the lowest countries were found among processed meat and fish products separately. Large sodium content variations were also found for certain specific food subcategories across the five countries, and across different food subcategories within each country.

Conclusion: Processed meat and fish products differ greatly in sodium content across different countries and different food subcategories. This indicates a great potential for sodium reduction through reformulation by food producers, and selection of less salted food by consumers.

Keywords: sodium, sodium reduction, processed foods, food reformulation, FoodSwitch

For peer review only

Strengths and limitations of this study

Strengths :

1) This study is the first time to conduct a cross-sectional survey of the sodium content of processed meat and fish products in supermarkets among five countries using global food composition database.

Potential limitations:

1) Products were obtained only in selected stores at a specific time point in each country.
2) We did not capture household consumer panel food-purchasing data to quantify actual sodium consumption of processed meat and fish products.

Introduction

High sodium intake is the major cause of high blood pressure and increases the risk of cardiovascular disease, renal disease and premature mortality.[1] Processed meat and fish products constitute important categories of processed food, providing high-quality protein, minerals and vitamins; and the processing itself offers an opportunity to add flavour, improve food safety and extend shelf-life. However, the high sodium content, which is known to be a key factor for the quality and sensory attributes of processed meat and fish, is usually of high health concern. The global average sodium intake was about 4000 mg/d in 2010, twice the maximum 2000 mg/d recommended by the World Health Organization (WHO).[2] A previous study conducted in 2013 in China reported that the average sodium content of processed meat and fish products was 1029 mg/100g and 1424 mg/100g respectively, amounting to over half of the recommended daily sodium intake.[3] Although in developing countries like China, sodium intake mainly derives from cooking, the consumption of processed foods including meat and fish products tends to increase with the rapid urbanization and

For peer review only

nutrition transition.[4] In developed countries where more than three quarters of sodium coming from processed foods, it was estimated that sodium intake from meat and meat products contributed approximately 16-25% of total daily sodium intake.[5] As such, with numerous countries endeavouring to reduce population sodium intake in response to the WHO goal of 30% sodium reduction by 2025, it is worth paying attention to the high sodium content of processed meat and fish products worldwide.[6]

Many countries have made efforts to reduce the sodium content of processed foods. For instance, the UK, US and Australia have set voluntary targets for sodium reduction in various categories of processed foods.[7-9] South Africa was the first country to include the statutory maximum sodium target in several processed food categories.[10] This target-based approach has been shown to be effective in reducing sodium content in many food products[11 12] and, for the same food category, the sodium level is much higher in the countries without sodium reduction target than those with the target.[4] A case in point is the sodium content of sauces in China vs UK. The median sodium contents were on average 4.4-fold greater in Chinese sauces compared with their UK equivalents.[13]

The George Institute for Global Health established a global food composition database in 2010 as part of The International Network for Food and Obesity/non-communicable diseases Research, Monitoring and Action Support (INFORMAS), with an aim to collate and track the nutritional compositions of processed foods worldwide. The global food composition database uses a standardized methodology for data collection and processing, with data available from more than ten countries as of 2020,[14-16] making the comparison of sodium content across countries possible. Using data from the George Institute global food composition database, the present study aims to compare the sodium content in processed meat and fish products across

For peer review only

five countries including the UK, US, Australia, South Africa and China, in order to find potential strategies to reduce the sodium content of these products.

Materials and Methods

Data Collection

Images of pre-packaged foods were taken using smartphone applications (The George Institute Data Collector and FoodSwitch)[14] by trained data collectors as well as consumers through crowdsourcing and uploaded to a central content management system. Professionally trained data entry clerks then entered the information displayed in products package, including product information, nutritional information and ingredients according to standard procedures. All entered information was reviewed by a second data entry clerk for accuracy. Products with verified information were classified according to a standard food categorization system. This study used data of processed meat and fish products collected in the UK, Australia, South Africa and China available within the George Institute global food composition database, with the data collection time ranging from 2012 to 2018. We also obtained processed meat and fish products data from the US, which were shared by Label Insight Inc. to The George Institute for non-profit research.

Data Categorisation

Within the food categorization system, processed meat products and processed fish products were two independent categories. Processed meat products were further classified to the following 16 subcategories: meat-free products, bacon, canned meat, frozen meat, meat burgers, salami and cured meats, sausage and hot dogs, sliced meat, dried meat, pate and meat spreads, kebabs, other meat products, raw flavoured meats, whole hams and similar products, roasted chicken, raw unflavoured meats. Processed

For peer review only

fish products were divided into 4 subcategories, including canned fish, chilled fish, frozen fish and other fish.

Data Exclusion Criteria

Products with no declaration of neither sodium nor salt values were excluded. For identical products with same sodium content in different package sizes, it is regarded as a duplicate product, only one product was included.

Data analysis

Sodium value data were obtained from the Nutrition Information Panel (NIP). For products with only salt values available, sodium values were calculated from salt values divided by 2.5. Median and interquartile range (IQR) were used to describe the distribution of sodium values (mg/100g) given the non-normal distribution of the data. The Kruskal-Wallis H test was used to compare differences in sodium values of processed meat and fish products across the five countries. If the difference was statistically significant, post-hoc tests were carried out using Bonferonni correction. The subcategory with data records equal to or less than 5 was excluded from the analysis for subcategory comparisons.

In reference to the "Traffic Light" criteria developed by the UK, sodium level was defined as low (< 120 mg/100g), medium (120 ≤ sodium ≤ 600 mg/100g), and high (> 600 mg/100g); and expressed as green, amber and red accordingly in a horizontal bar chart to show the sodium contents visually.[17] The 2017 UK sodium reduction targets were used to assess the percentage of products reaching the targets across the five countries.[18] The maximum sodium targets of each category were selected for ease of comparison, and the average targets were used where maximum targets were not provided. The Chi-square tests were used to compare the proportion of products that meet the 2017 UK sodium reduction targets.

For peer review only

Moreover, daily sodium intake from each serving of meat or fish products were compared with the WHO maximum sodium recommendation (2000 mg/d) to further measure the sodium burden due to the consumption of processed meat or fish products. According to previous studies in Australia, the average serving size of meat products was 94 g.[19] For simplicity, the present study used 100g as the serving size of meat and fish products. The percentage contribution of sodium intake from each serving of meat or fish products towards the recommended daily sodium intake was coloured into red, yellow, and green respectively to represent if the percentage is in the upper (>66%), middle (>33%, ≤66%) and lower (≤33%) range.

A two-sided p-value of less than 0.05 was considered significant in the statistical tests. The analyses were conducted using Stata/SE 14.2 and IBM SPSS 21.0.

Patient and Public Involvement

No patient involved.

Results

A total of 33955 processed meat and fish products were collected from the five countries, of which 7455(21.96%) were excluded because of missing sodium data or duplicate products, leaving 26500 (78.04%) products for analysis in this study (Figure 1). The total number of products per country ranged from 885 for the UK to 17098 for the US (Table 1). The number of products per category ranged from 1 in meat-free products, kebabs and roasted chicken to 2817 in sausages and hot dogs.

For peer review only

Table 1 Sodium Content of Processed Meat and Fish Products across Five Countries (mg/100g)

	USA (n=17098)			SA (n=946)			Australia (n=5673)			UK (n=885)			China (n=1898)			*K-W H TEST*
	n	median	IQR	n	median	IQR	n	median	IQR	n	median	IQR	n	median	IQR	*p*-Value
**Total**	17098	655	353-981	946	571	362-876	5673	489	335-854	885	432	236-786	1898	1050	774-1473	<0.001
**Meat**	12954	768	474-1071	612	754	518-1020	3836	580	376-990	558	590	275-904	1641	1066	800-1450	<0.001
meat-free products	372	478	386-607	—	—	—	—	—	—	10	413	236-550	1	1102	1102	0.175
bacon	707	1667	1050-1857	36	1018	823-1155	289	1150	1020-1700	43	1612	1140-2162	33	805	750-1000	<0.001
canned meat	462	607	446-964	44	659	517-855	127	717	483-900	8	275	236-826	48	762	688-887	0.018
frozen meat	1169	532	400-690	123	460	347-577	875	440	347-560	86	275	236-354	33	160	64-713	<0.001
meat burgers	824	476	305-647	47	638	500-794	162	475	390-584	6	393	315-472	7	612	486-703	<0.001
salami and cured meats	554	1607	1357-1750	25	1633	1415-1838	265	1410	1200-1600	5	1573	1376-1612	115	1200	949-1532	<0.001
sausage and hot dogs	2817	829	696-974	133	814	684-935	456	704	565-900	36	550	452-747	269	991	832-1111	<0.001
sliced meat	1937	875	750-1088	84	900	745-1100	359	989	816-1100	173	668	590-865	23	1132	845-1250	<0.001
dried meat	1383	1536	1036-1929	43	2144	1682-2280	126	1760	1400-2000	—	—	—	281	1509	1153-1760	<0.001
pate and meat spreads	83	679	518-911	14	789	438-861	89	480	310-603	28	629	550-708	5	1916	1670-2490	<0.001
kebabs	2	493	462-525	—	—	—	38	408	294-504	1	393	393	—	—	—	—
other meat products	427	589	94-1071	26	865	560-1070	83	570	340-925	13	275	236-472	649	1050	782-1390	<0.001
raw flavoured meats	501	446	254-750	24	465	356-580	678	368	245-502	27	197	197-315	42	563	382-763	<0.001
whole hams and similar products	4	848	750-1518	2	839	744-934	80	1080	1000-1400	12	1081	983-1356	103	1039	940-1250	0.977
roasted chicken	2	563	357-768	1	415	415	37	359	271-548	3	197	118-236	9	893	693-996	<0.001
raw unflavoured meats	1710	71	63-402	10	69	58-120	172	66	50-323	62	79	79	23	122	70-234	0.011
**Fish**	4144	364	208-529	334	356	265-453	1837	395	286-540	327	354	236-550	257	942	470-1867	<0.001
canned fish	1219	388	299-467	168	353	280-400	821	380	309-472	66	354	315-393	138	902	599-1586	<0.001
chilled fish	147	389	171-691	36	449	226-798	332	587	324-917	126	511	157-747	53	1744	370-5072	<0.001
frozen fish	2733	347	152-541	100	295	169-434	559	340	225-449	117	275	197-354	36	131	73-715	<0.001
other fish	45	5389	3813-6000	30	451	362-580	125	860	426-4990	18	550	432-747	30	1305	1147-1644	<0.001

For peer review only

Levels of Sodium Content for Processed Meat and Fish across the Five Countries

Table1 showed the sodium content of processed meat and fish products across the five countries. Overall, for processed meat and fish products combined, China had the highest sodium level(1050 mg/100g, IQR: 774-1473), ranking as the country with the saltiest products for both meat (1066 mg/100g, IQR: 800-1450) and fish products (942 mg/100g, IQR: 470-1867) , followed by the US, South Africa, Australia, and the UK (432 mg/100g, IQR: 236-786). Taking meat products alone, Australia had lower median sodium content (580 mg/100g, IQR: 376-990) than the UK (590 mg/100g, IQR: 275-904). Significant differences in sodium levels were seen in 18 subcategories among five countries. For example, the sodium content of roasted chicken in China was 4.5 times that of the UK (893 mg/100g vs 197 mg/100g); chilled fish in China, 4.5 times that of the US (1744 mg/100g vs 389 mg/100g); pate and meat spreads in China, about 4 times that of Australia (1916 mg/100g vs 480 mg/100g). However, the sodium content of bacon, frozen meat, salami and cured meats, dried meat and frozen fish in China was the lowest among five countries. Taking bacon as an example, the median sodium contents ordered from highest to lowest were the US (1667 mg/100g), the UK (1612 mg/100g), Australia (1150 mg/100g), South Africa (1018 mg/100g) and China (805 mg/100g). Within each country, the sodium content also varied greatly across different subgroups with raw unflavoured meats being the lowest sodium content subcategory.

Comparison of Sodium Content Using Traffic Light Criteria

Across the five countries, a large part of processed meat and fish products fell into the red and amber category, the highest proportion of green light was in the UK, accounting for 12.66% of the meat and fish products. China had the largest proportion of red light (85.83%) and the smallest proportion of green light (3.64%) ($\chi 2$=1101.13,

For peer review only

p<0.001). A similar differences was seen in processed meat products ($\chi 2$=774.95, p<0.001). For processed fish products, the highest green light was 14.50% in the US, followed by 12.84% in the UK, and South Africa had the largest portion of amber light (84.73%) and the lowest red light (8.68%) and green light (6.59%)($\chi 2$=277.49, p<0.001).(Figure 2-1,2-2,2-3)

Comparison of Sodium Content to 2017 UK Sodium Reduction Targets

Of the 13 categories of processed meat and fish products with 2017 UK sodium reduction targets, the countries with average sodium contents reaching 2017 UK sodium reduction targets from high to low were the UK (26.6%), Australia (23.2%), South Africa (22.4%), the US (18.4%) and China (7.1%). Statistically significant differences were observed in the selected food categories among countries (p<0.001 for bacon, canned meat, frozen meat, meat burgers, sausage and hot dogs, other meat products and canned fish). The UK had the highest percentage of achieving the targets except in the bacon category, only 14.0% of bacon in the UK reached the target, lower than the US (28.2%), Australia (50.2%), SA (75.0%) and China (84.9%). (Table 2)

For peer review only

Table 2 No. and percentage of products with sodium content meeting 2017 UK Sodium Targets

Categories	2017 UK Sodium Targets (mg/100g)	China n (%)	UK n (%)	Australia n (%)	SA n (%)	US n (%)	*p*-Value
Meat-Free Products	500	0(0.0)	5(50.0)	—	—	212(57.0)	0.907
Bacon[1]	1152	28(84.9)	6(14.0)	145(50.2)	27(75.0)	199(28.2)	<0.001
Canned Meat	272	0(0.0)	4(50.0)	5(3.9)	0(0.0)	24(5.2)	<0.001
Frozen Meat[1]	272	17(51.5)	36(41.9)	87(9.9)	18(14.6)	139(11.9)	<0.001
Meat Burgers	352	1(14.3)	2(33.3)	27(16.7)	4(8.5)	249(30.2)	<0.001
Salami and Cured Meats[1]	652	5(4.4)	0(0.0)	2(0.8)	0(0.0)	18(3.3)	0.08
Sausage and Hot Dogs	600	7(2.6)	21(58.3)	147(32.2)	14(10.5)	350(12.4)	<0.001
Sliced Meat	272	1(4.4)	5(2.9)	9(2.5)	0(0.0)	32(1.7)	0.218
Kebabs	352	—	0(0.0)	14(36.8)	—	0(0.0)	—
Other Meat Products	300	29(4.5)	8(61.5)	17(20.5)	2(7.7)	150(35.1)	<0.001
Whole Hams and Similar Products[1]	652	0(0.0)	1(8.3)	3(3.8)	0(0.0)	0(0.0)	0.036
Roasted Chicken	272	0(0.0)	3(100.0)	10(27.0)	0(0.0)	0(0.0)	0.189
Canned Fish[1]	360	13(9.4)	44(66.7)	367(44.7)	89(53.0)	554(45.5)	<0.001
Total/Average		101(7.1)	135(26.6)	833(23.2)	154(22.4)	1927(18.4)	<0.001

[1]Average Sodium Targets. The maximum sodium targets of each category were selected for ease of comparison, and the average targets were used where maximum target was not provided.

Contribution of Sodium Content Per Serving Product to WHO Daily Sodium Intake Recommendation

According to Table 3, consumption of one serving size of meat (100 g/serving) or fish products (100 g/serving) in China will account for 47.2% of the WHO recommended maximum daily intake (2000 mg/d), nearly half of the daily intake. If the consumed products happen to be pate and meat spreads, then the sodium intake per serving product will contribute to 95.8% of daily sodium upper limit. The contribution to daily sodium intake from each serving meat and fish products are

For peer review only

47.1% in the US, 36.9% in South Africa, 34.6% in Australia and 27.1% in the UK. While consumption of a serving bacon or salami in the UK will contribute to 80.6% or 78.7% of daily sodium limit respectively, more than two thirds of the daily intake.

Table 3 Contribution (%)[1] towards the WHO daily intake recommendation (2000 mg/d) for each serving (100g) consumption of processed meat and fish products

Categories	China	UK	Australia	SA	US
Meat-free products	55.1	20.7	—	—	23.9
Bacon	40.3	80.6	57.5	50.9	83.4
Canned meat	38.1	13.8	35.9	33.0	30.4
Frozen meat	8.0	13.8	22.0	23.0	26.6
Meat burgers	30.6	19.7	23.8	31.9	23.8
Salami and cured meats	60	78.7	70.5	81.7	80.4
Sausage and hot dogs	49.6	27.5	35.2	40.7	41.5
Sliced meat	56.6	33.4	49.5	45.0	43.8
Dried meat	75.5	—	88.0	107.2	76.8
Pate and meat spreads	95.8	31.5	24.0	39.5	34.0
Kebabs	—	19.7	20.4	—	24.7
Other meat products	52.5	13.8	28.5	43.3	29.5
Raw flavoured meats	28.2	9.9	18.4	23.3	22.3
Whole hams and similar products	52.0	54.1	54.0	42.0	42.4
Roasted chicken	44.7	9.9	18.0	20.8	28.2
Raw unflavoured meats	6.1	4.0	3.3	3.5	3.6
Canned fish	45.1	17.7	19.0	17.7	19.4
Chilled fish	87.2	25.6	29.4	22.5	19.5
Frozen fish	6.6	13.8	17.0	14.8	17.4
Other fish	65.3	27.5	43.0	22.6	269.5
Average	47.2	27.1	34.6	36.9	47.1

[1]Contribution (%) towards the WHO target = Median sodium / 2000*100%. The percentage contribution of sodium intake from each serving of meat or fish products towards the recommended daily sodium intake was coloured into red, yellow, and green respectively to represent if the percentage is in the upper (>66%), middle (>33%, ≤66%) and lower (≤33%) .

Discussion

This study provided the first detailed evaluation of sodium content in processed meat and fish products among five countries. The results showed extremely wide discrepancy within and between countries. Overall, processed meat and fish products in the UK had the lowest median sodium content, and China had the highest sodium in both meat and fish products. The sodium content of meat and fish products in each country was high compared with "Traffic Light" criteria with only 10% of the products falling into the green light group in the UK and US and no more than 5% in China and South Africa. The percentage of products meeting 2017 UK sodium reduction targets were generally low ranging from 7.1% (China) to 26.6% (the UK). A 100 g serving size of processed meat and fish products could averagely contribute to one half/third of WHO daily maximum sodium intakes in all countries.

In developed countries like Australia, US and the UK, processed foods provide 75%~80% of sodium intake.[20 21] It was reported that processed meat products accounted for about 20% of daily meat consumption and contributed to around 10% daily sodium intake in Australia.[11] In South Africa, processed meat was also a major sodium source other than bread among processed foods which contributed to about 50% of sodium intake.[21] In China, where 70%~80% of sodium came from cooking at home, with a remarkable increase in consumption of processed foods and meals out of home in recent years, sodium intake from meat and fish products is an emerging concern.[22] Our findings of the very high sodium levels in processed meat and fish products across all the five countries, clearly indicates that a reduction in the sodium content of these products would help reduce population sodium intake.

One strategy to reduce sodium intake from meat and fish products would be to replace high-sodium products with low-sodium products. For example, choosing raw

For peer review only

unflavoured meats instead of salami and cured meats would decrease ten to twenty times of sodium intake in all countries. However, different subcategories of meat and fish products have distinct different organoleptic properties, which coupled with the convenience of pre-prepared meat products are usually the drivers for consumers' choice.[23] Therefore, reducing sodium in all meat and fish products would be the optimal strategy. The huge discrepancy of sodium content in the same subcategory within and between the countries indicated the big potential of sodium reduction through reformulation of meat and fish products. Additionally, the comparison of sodium contents across the countries with different sodium reduction policies in meat and fish products suggested that setting sodium targets for processed foods would be an effective way to reduce sodium contents of packaged foods, which is in alignment with many other studies.[4 11 13 24]

This study showed China had on average the saltiest meat and fish products among five countries, which is likely to be due to the lack of sodium targets to limit the sodium added to the products. The other four countries, the UK, the US, Australia and South Africa, all have set voluntary or mandatory sodium targets for meat and fish products along within a comprehensive sodium reduction policy/program in these countries. The UK had issued four sets of voluntary sodium targets for over 80 categories of processed foods since 2006 and had set up a successful sodium reduction model for other countries through this incremental sodium reduction strategy.[7] Following the UK, the US and Australia set the voluntary sodium targets for various processed foods through the National Salt Reduction Initiative in 2009 in the US and the Food and Health Dialogue in 2010 in Australia, respectively.[20 25] South Africa became the first country to regulate legislated sodium limits for a range of food products in 2012.[12] The results of comparing the latest 2017 UK sodium reduction

For peer review only

targets were consistent with the median meat and fish products showing the highest proportion of meeting the targets in the UK, followed by Australia, South Africa, US and China, which to a certain extent reflected the implementation of sodium reduction policy.

Our results also showed that the proportion of products that met the sodium reduction targets was low across all the countries with no more than 30% below the targets in the UK, which was much lower than the 90% and 70% of noodles and sauces meeting the 2017 UK sodium reduction targets in the UK.[4, 13] Some subcategories of meat products such as bacon even had the highest sodium content in the UK among five countries, suggesting robust implementation and monitoring of the voluntary targets are still needed to sustain the sodium reduction results in the UK and the like countries with sodium targets in place. Moreover, the 2017 UK sodium reduction targets were more rigorous compared with that of other countries. Other studies comparing the sodium contents against the country-specific sodium showed higher proportion with about half of meat products meeting the individual targets in Australia, South Africa and the US.[11, 12, 20] It was therefore worth learning for China to take into account both the technical feasibility and consumer acceptability if sodium targets were to be set in the future. Front-of-Pack labelling such as the "Traffic Light" labelling in the UK and the Health Star Rating in Australia as well as consumer awareness campaigns may increase consumer acceptability and demand for healthier products.[21, 25]

A key strength of the present study is that this is the first time to conduct a cross-sectional survey of the sodium content of processed meat and fish products in supermarkets among five countries using global food composition database. The standardized methods for data collection and processing, including standardized food

For peer review only

categorization, ensured the comparability of the data. Potential limitations should also be considered. First, products were obtained only in selected stores at a specific time point in each country. But the selected stores were major supermarket chains with a large market share in each country and is likely to represent a large part of products available within the countries. Second, we did not capture household consumer panel food-purchasing data to quantify actual sodium consumption of processed meat and fish products. Although the crowdsourcing element of the data collection may in part reflect what consumers are eating, future studies should consider using proper product sales data or consumption data to estimate the actual sodium intake from processed meat and fish products in each country.

Conclusions

The sodium content of meat and fish products in all the selected countries was very high with a 100 g serving size of meat and fish products contributing to one half/third of WHO recommended maximum daily sodium intake. There were big variations within and between the five countries with different sodium reduction policies, which implies great potential of sodium reduction in meat and fish products by setting feasible sodium reduction targets in countries without sodium reduction program and sustaining robust implementation and monitoring of the targets in the countries with sodium targets in place, as well as selection of less salted food by consumers.

Acknowledgments

We thank Prof Feng J He, Monique Tan, Prof Graham MacGregor from Queen Mary University of London provided data of the UK, Prof Bruce Neal from The George Institute for Global Health permitted and coordinated the data sharing. Blaire Jesseph from Label Insight in the US and Terry Harris from Vitality South Africa collaborated

For peer review only

with The George Institute in developing the dataset of the US and South Africa, respectively. Fraser Taylor, Kylie Howes and Lizzy Dunford from The George Institute FoodSwitch team mapped the data and directly provided data of Australia, South Africa and US. We appreciate Jimmy Louie from The University of Hong Kong for his contribution to the revision of the very first draft.

The authors' contributions were as follows-Yuzhu Song, Yuan Li, Chunlei Guo, Puhong Zhang and Yuxia Ma: designed the study; Liping Huang, Monique Tan, Feng J He, Terry Harris and Graham MacGregor: provided the data; Yuzhu Song, Yishan Wang, Jingmin Ding and Le Dong: analyzed the data; Yuzhu Song: prepared the first draft of the manuscript; Yuan Li, Chunlei Guo, Puhong Zhang and Yuxia Ma: made critical revisions to the manuscript for important intellectual content; and all authors reviewed and approved the final draft. None of the authors reported a conflict of interest related to this study.

Funding: This research was funded by National Key R&D Program of China, grant number 2016YFC1300200 and the National Institute for Health Research (NIHR, NIHR Global Health Research Unit Action on Salt China at Queen Mary University of London) using Official Development Assistance (ODA) funding (16/136/77). The views expressed in this publication are those of the author(s) and not necessarily those of the funders.

For peer review only

References

1. Forouzanfar MH, Alexander L, Anderson HR, et al. Global, regional, and national comparative risk assessment of 79 behavioural, environmental and occupational, and metabolic risks or clusters of risks in 188 countries, 1990–2013: a systematic analysis for the Global Burden of Disease Study 2013. The Lancet 2015;386(10010):2287-323. doi: 10.1016/s0140-6736(15)00128-2

2. Powles J, Fahimi S, Micha R, et al. Global, regional and national sodium intakes in 1990 and 2010: a systematic analysis of 24 h urinary sodium excretion and dietary surveys worldwide. BMJ Open 2013;3(12)

3. Huang L, Neal B, Dunford E, et al. Completeness of nutrient declarations and the average nutritional composition of pre-packaged foods in Beijing, China. Preventive medicine reports 2016;4:397-403.

4. Clare F, Karen C, Michelle C, et al. Know Your Noodles! Assessing Variations in Sodium Content of Instant Noodles across Countries. Nutrients 2017;9(6):612.

5. World Health Organization. *Diet, nutrition and the prevention of chronic disease: WHO Technical Report.* Geneva: World Health Organization, 2013.

6. World Health Organization. *Global Action Plan for the Prevention and Control of Noncommunicable Diseases 2013–2020.* Geneva: World Health Organization, 2013.

7. He FJ, Brinsden H, Macgregor GA. Salt reduction in the United Kingdom: a successful experiment in public health. *Journal of Human Hypertension* 2014;28(6):345-52.

8. Lindberg R, Nichols T, Yam C. The Healthy Eating Agenda in Australia. Is Salt a Priority for Manufacturers? *Nutrients* 2017;9(8):881-.

9. Reeve B, Magnusson R. Food reformulation and the (neo)-liberal state: new strategies for strengthening voluntary salt reduction programs in the UK and USA.

For peer review only

Public Health 2015;129(4):1061-73.

10. Webster J, Crickmore C, Charlton K, et al. South Africa's salt reduction strategy: Are we on track, and what lies ahead? *S Afr Med J* 2016;107(1):20-21.

11. Emalie, Sparks, Clare, et al. Sodium Levels of Processed Meat in Australia: Supermarket Survey Data from 2010 to 2017. *Nutrients* 2018

12. Peters S, Dunford E, Ware L, et al. The Sodium Content of Processed Foods in South Africa during the Introduction of Mandatory Sodium Limits. *Nutrients* 2017;9(4) doi: 10.3390/nu9040404

13. Tan M, He FJ, Ding J, et al. Salt content of sauces in the UK and China: cross-sectional surveys. *BMJ Open* 2019;9(9):e025623.

14. Neal B, Sacks G, Swinburn B, et al. Monitoring the levels of important nutrients in the food supply. *Obesity Reviews* 2013;14:49-58.

15. Dunford E. International collaborative project to compare and track the nutritional composition of fast foods. *BMC Public Health* 2012;12(559):559-59.

16. Antoine M, Veli-Matti L, Johan S,et al. Measuring the healthiness of child-targeted cereals: a study of the FoodSwitch platform in Sweden. *JMIR* Published Online First:13 January 2020 doi: doi.org/10.2196/preprints.17780).

17. Freire WB, Waters WF, Rivas-Mario G, et al. A qualitative study of consumer perceptions and use of traffic light food labelling in Ecuador. *Public Health Nutrition* 2017;20(5):1-9.

18. Salt Reduction Targets for 2017. *Public Health England.* https://assets.publishing.service.gov.uk/government/uploads/system/uploads/attachment_data/file/604338/Salt_reduction_targets_for_2017.pdf (accessed on 9 September 2020).

19. Crino M, Sacks G, Dunford E, et al. Measuring the Healthiness of the Packaged

For peer review only

Food Supply in Australia. *Nutrients* 2018;10(6):702.

20. Curtis CJ, Clapp J, Niederman SA, et al. US Food Industry Progress During the National Salt Reduction Initiative: 2009–2014. *American Journal of Public Health* 2016;106(10):1815-19.

21. Charlton KE, Webster J, Kowal P. To Legislate or Not to Legislate? A Comparison of the UK and South African Approaches to the Development and Implementation of Salt Reduction Programs. *Nutrients* 2014;6(9):3672-95.

22. Shao S, Hua Y, Yang Y, et al. Salt reduction in China: a state-of-the-art review. *Risk Management and Healthcare Policy* 2017;10:17-28.

23. Nam KC, Jo C, Lee M. Meat products and consumption culture in the East. *Meat Science* 2010;86(1):95-102.

24. Eyles H, Webster JL, Jebb SA, et al. Impact of the UK voluntary sodium reduction targets on the sodium content of processed foods from 2006 to 2011: Analysis of household consumer panel data. *Preventive Medicine* 2013;57(5):555-60.

25. Webster J, Trieu K, Dunford E, et al. Salt reduction in Australia: from advocacy to action. *Cardiovascular diagnosis and therapy* 2015;5(3):207-18.

For peer review only

Figure 1 Flow diagram of product selection.

(CN: China; UK: The United Kingdom; AU: Australia; SA: South Africa; US: The United States.)

Figure 2-1 Sodium content Traffic Light on processed meat and fish products among five countries

Figure legend:

■ Red (High)

■ Amber (Medium)

■ Green (Low)

Figure 2-2 Sodium content Traffic Light on processed meat products among five countries

Figure legend:

■ Red (High)

■ Amber (Medium)

■ Green (Low)

Figure 2-3 Sodium content Traffic Light on processed fish products among five countries

Figure legend:

■ Red (High)

■ Amber (Medium)

■ Green (Low)

For peer review only

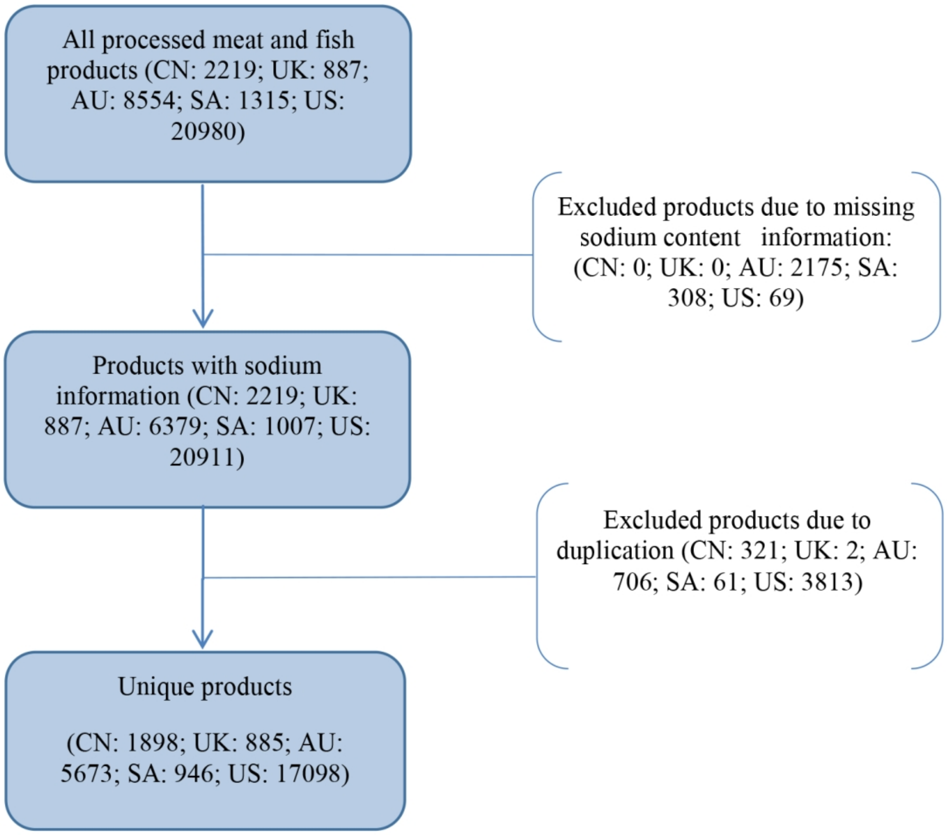

Figure 1 Flow diagram of product selection.

CN: China; UK: The United Kingdom; AU: Australia; SA: South Africa; US: The United States.

Figure 1 Flow diagram of product selection.
CN: China; UK: The United Kingdom; AU: Australia; SA: South Africa; US: The United States.

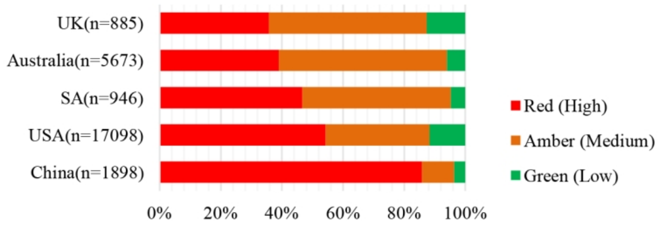

Figure 2-1 Sodium content Traffic Light on processed meat and fish products among five countries

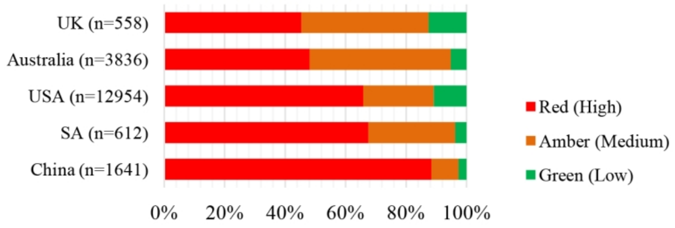

Figure 2-2 Sodium content Traffic Light on processed meat products among five countries

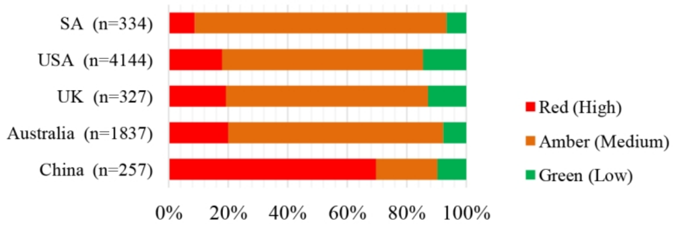

Figure 2-3 Sodium content Traffic Light on processed fish products among five countries

Figure 2-1 Sodium content Traffic Light on processed meat and fish products among five countries;
Figure 2-2 Sodium content Traffic Light on processed meat products among five countries;
Figure 2-3 Sodium content Traffic Light on processed fish products among five countries;

Dear Editors,

R ”, BMJ Open”.

This study aims to compare the sodium content in processed meat and fish products among five countries and provide a basis for feasible strategies of sodium reduction in such products. Nutrition information of 19601 meat and 6899 fish products was collected using the FoodSwitch mobile application from China, the United Kingdom (UK), Australia, South Africa and the United States (US) from 2012 to 2018 and analyzed for cross-sectional comparisons.

To our knowledge, this study is the first time to conduct a cross-sectional survey of the sodium content of processed meat and fish products in supermarkets among five countries using the FoodSwitch application with a large and wide range of data. The standardized methods for data collection and processing, including standardized food categorization, ensured the comparability of the data.

And we found that the very high sodium levels in processed meat and fish products across all the five countries, clearly indicates that a reduction in the sodium content of these products would help reduce population sodium intake. Additionally, the comparison of sodium contents across the countries with different sodium reduction policies in meat and fish products confirmed that setting sodium targets for processed foods is an effective way to reduce sodium contents of packaged foods, and provided a basis for feasible strategies of sodium reduction in such products.

For peer review only

Here, we confirm that this is an original research, and neither the manuscript nor any parts of its content are currently under consideration or published in another journal. The authors declare no conflict of interest.

Finally, I have a little doubt that my article involves two corresponding authors. I don't know if your magazine is considering publishing such articles.

Thank you for your consideration. We look forward to hearing from you.

I y y q , ’ e address below.

Yours sincerely

Yuzhu Song

E-mail: 1493745867@qq.com

Tel: +86-17330056895

Institution:

Hebei Medical University

Address:

361 Zhongshan East Road, Chang'an District, Shijiazhuang City, Hebei Province 050017, China

For peer review only

STROBE Statement—Checklist of items that should be included in reports of ***cross-sectional studies***

	Item No	Recommendation	Page No
**Title and abstract**	1	(*a*) Indicate the study's design with a commonly used term in the title or the abstract	P1
		(*b*) Provide in the abstract an informative and balanced summary of what was done and what was found	P3
**Introduction**			
Background/rationale	2	Explain the scientific background and rationale for the investigation being reported	P4
Objectives	3	State specific objectives, including any prespecified hypotheses	P5
**Methods**			
Study design	4	Present key elements of study design early in the paper	P5-6
Setting	5	Describe the setting, locations, and relevant dates, including periods of recruitment, exposure, follow-up, and data collection	P5-6
Participants	6	(*a*) Give the eligibility criteria, and the sources and methods of selection of participants	P6
Variables	7	Clearly define all outcomes, exposures, predictors, potential confounders, and effect modifiers. Give diagnostic criteria, if applicable	NA
Data sources/ measurement	8*	For each variable of interest, give sources of data and details of methods of assessment (measurement). Describe comparability of assessment methods if there is more than one group	P6
Bias	9	Describe any efforts to address potential sources of bias	P5-6
Study size	10	Explain how the study size was arrived at	P6
Quantitative variables	11	Explain how quantitative variables were handled in the analyses. If applicable, describe which groupings were chosen and why	NA
Statistical methods	12	(*a*) Describe all statistical methods, including those used to control for confounding	P6-7
		(*b*) Describe any methods used to examine subgroups and interactions	NA
		(*c*) Explain how missing data were addressed	P6
		(*d*) If applicable, describe analytical methods taking account of sampling strategy	P7
		(*e*) Describe any sensitivity analyses	NA
**Results**			
Participants	13*	(a) Report numbers of individuals at each stage of study—eg numbers potentially eligible, examined for eligibility, confirmed eligible, included in the study, completing follow-up, and analysed	P8
		(b) Give reasons for non-participation at each stage	P8
		(c) Consider use of a flow diagram	NA
Descriptive data	14*	(a) Give characteristics of study participants (eg demographic, clinical, social) and information on exposures and potential confounders	NA
		(b) Indicate number of participants with missing data for each variable of interest	NA
Outcome data	15*	Report numbers of outcome events or summary measures	NA
Main results	16	(*a*) Give unadjusted estimates and, if applicable, confounder-adjusted estimates and their precision (eg, 95% confidence interval). Make clear which confounders were adjusted for and why they were included	P10

For peer review only - http://bmjopen.bmj.com/site/about/guidelines.xhtml

		(*b*) Report category boundaries when continuous variables were categorized	NA
		(*c*) If relevant, consider translating estimates of relative risk into absolute risk for a meaningful time period	NA
Other analyses	17	Report other analyses done—eg analyses of subgroups and interactions, and sensitivity analyses	P10-13
**Discussion**			
Key results	18	Summarise key results with reference to study objectives	P14-16
Limitations	19	Discuss limitations of the study, taking into account sources of potential bias or imprecision. Discuss both direction and magnitude of any potential bias	P17
Interpretation	20	Give a cautious overall interpretation of results considering objectives, limitations, multiplicity of analyses, results from similar studies, and other relevant evidence	P17
Generalisability	21	Discuss the generalisability (external validity) of the study results	P17
**Other information**			
Funding	22	Give the source of funding and the role of the funders for the present study and, if applicable, for the original study on which the present article is based	P18

*Give information separately for exposed and unexposed groups.

Note: An Explanation and Elaboration article discusses each checklist item and gives methodological background and published examples of transparent reporting. The STROBE checklist is best used in conjunction with this article (freely available on the Web sites of PLoS Medicine at http://www.plosmedicine.org/, Annals of Internal Medicine at http://www.annals.org/, and Epidemiology at http://www.epidem.com/). Information on the STROBE Initiative is available at www.strobe-statement.org.

For peer review only

Cross-sectional comparisons of sodium content in processed meat and fish products among five countries - potential for feasible targets and reformulation

Journal:	*BMJ Open*
Manuscript ID	bmjopen-2020-046412.R1
Article Type:	Original research
Date Submitted by the Author:	25-Feb-2021
Complete List of Authors:	Song, Yuzhu; Hebei Medical University,<br>Li, Yuan; The George Institute for Global Health, Peking University Health Science Centre; Faculty of Medicine, University of New South Wales, Australia<br>Guo, Chunlei; The George Institute for Global Health, Peking University Health Science Centre<br>Wang, Yishan; University of Hong Kong<br>Huang, Liping; The George Institute for Global Heath, Food Policy<br>Tan, Monique; Wolfson Institute of Preventive Medicine, Barts and The London School of Medicine & Dentistry, Queen Mary University of London, Centre for Environmental and Preventive Medicine<br>He, Feng; Queen Mary University of London, Wolfson Institute of Preventive Medicine<br>Harris, Terry; Discovery Vitality, Johannesburg, South Africa<br>MacGregor, Graham; Queen Mary University of London, Wolfson Institute of Preventive Medicine<br>Ding, Jingmin; The George Institute for Global Health, Peking University Health Science Centre<br>Dong, Le; The George Institute for Global Health, Peking University Health Science Centre<br>Liu, Yu; Beihang University, School of Computing<br>Wang, Huijun; Chinese Center for Disease Control and Prevention National Institute for Nutrition and Health<br>Zhang, Puhong ; The George Institute at Peking University Health Science Center, Diabetes Program; Faculty of Medicine, University of New South Wales, Australia<br>Ma, Yuxia; Hebei Medical University
**Primary Subject Heading**:	Public health
Secondary Subject Heading:	Global health, Health policy, Public health
Keywords:	EPIDEMIOLOGY, Health policy < HEALTH SERVICES ADMINISTRATION & MANAGEMENT, Public health < INFECTIOUS DISEASES, PREVENTIVE MEDICINE

SCHOLARONE™
Manuscripts

I, the Submitting Author has the right to grant and does grant on behalf of all authors of the Work (as defined in the below author licence), an exclusive licence and/or a non-exclusive licence for contributions from authors who are: i) UK Crown employees; ii) where BMJ has agreed a CC-BY licence shall apply, and/or iii) in accordance with the terms applicable for US Federal Government officers or employees acting as part of their official duties; on a worldwide, perpetual, irrevocable, royalty-free basis to BMJ Publishing Group Ltd ("BMJ") its licensees and where the relevant Journal is co-owned by BMJ to the co-owners of the Journal, to publish the Work in this journal and any other BMJ products and to exploit all rights, as set out in our licence.

The Submitting Author accepts and understands that any supply made under these terms is made by BMJ to the Submitting Author unless you are acting as an employee on behalf of your employer or a postgraduate student of an affiliated institution which is paying any applicable article publishing charge ("APC") for Open Access articles. Where the Submitting Author wishes to make the Work available on an Open Access basis (and intends to pay the relevant APC), the terms of reuse of such Open Access shall be governed by a Creative Commons licence – details of these licences and which Creative Commons licence will apply to this Work are set out in our licence referred to above.

Other than as permitted in any relevant BMJ Author's Self Archiving Policies, I confirm this Work has not been accepted for publication elsewhere, is not being considered for publication elsewhere and does not duplicate material already published. I confirm all authors consent to publication of this Work and authorise the granting of this licence.

For peer review only

Cross-sectional comparisons of sodium content in processed meat and fish products among five countries - potential for feasible targets and reformulation

Corresponding authors: Puhong Zhang: The George Institute for Global Health at Peking University Health Science Center ; Faculty of Medicine, University of New South Wales, Australia; zpuhong@georgeinstitute.org.cn; and Yuxia Ma: Hebei Medical University; mayuxia@hebmu.edu.cn;

Co-authors:

1 Yuzhu Song: Department of nutrition and food hygiene; Hebei Medical University; Shijiazhuang; China.

2 Yuan Li : The George Institute for Global Health at Peking University Health Science Center; Beijing; China, and Faculty of Medicine, University of New South Wales, Australia.

3 Chunlei Guo: The George Institute for Global Health at Peking University Health Science Center; Beijing; China.

4 Yishan Wang : School of Public Health, LKS Faculty of Medicine, The University of Hong Kong, Hong Kong, China.

5 Liping Huang : The George Institute for Global Health | AUSTRALIA; Australia.

6 Monique Tan : Wolfson Institute of Preventive Medicine, Barts and The London School of Medicine & Dentistry, Queen Mary University of London, London, UK.

7 Feng J He : Wolfson Institute of Preventive Medicine, Barts and The London School of Medicine & Dentistry, Queen Mary University of London, London, UK.

8 Terry Harris : Discovery Vitality, Johannesburg, South Africa.

For peer review only

9 Graham A MacGregor : Wolfson Institute of Preventive Medicine, Barts and The London School of Medicine & Dentistry, Queen Mary University of London, London, UK.

10 Jingmin Ding: The George Institute for Global Health at Peking University Health Science Center; Beijing; China.

11 Le Dong: The George Institute for Global Health at Peking University Health Science Center; Beijing; China.

12 Yu Liu : School of Computer Science & Engineering, Beihang University; Beijing; China.

13 Huijun Wang: National Institute for Nutrition and Health at Chinese Center for Disease Control and Prevention; Beijing; China.

14 Puhong Zhang: The George Institute for Global Health at Peking University Health Science Center; Beijing; China, and Faculty of Medicine, University of New South Wales, Australia

15 Yuxia Ma: Department of nutrition and food hygiene; Hebei Medical University; Shijiazhuang; China.

Word count:3742

For peer review only

ABSTRACT

Introduction: Reducing sodium intake has been identified as a highly cost-effective strategy to prevent and control of high blood pressure and reduce cardiovascular mortality. This study aims to compare the sodium content in processed meat and fish products among five countries, which will contribute to the evidence-base for feasible strategies of sodium reduction in such products.

Methods: Sodium content on product labels of 26500 prepackaged products, 19601 meat and 6899 fish, was collected in supermarkets from five countries using the FoodSwitch mobile application from 2012 to 2018. To be specific, it was 1898 products in China, 885 in the United Kingdom (UK), 5673 in Australia, 946 in South Africa and 17098 in the United States (US). Cross-sectional comparisons of sodium levels and proportions meeting 2017 UK sodium reduction targets were conducted using Kruskal-Wallis H and the Chi-Square test respectively across the five countries.

Results: The results showed that processed meat and fish products combined in China had the highest sodium level (median 1050mg/100g, interquartile range [IQR]: 774-1473), followed by the US, South Africa, Australia, with the lowest levels found in UK (432mg/100g, IQR: 236-786) ($p<0.001$). Similar variations, i.e. a 2-3-fold difference of sodium content between the highest and the lowest countries were found among processed meat and fish products separately. Large sodium content variations were also found in certain specific food subcategories across the five countries, as well as across different food subcategories within each country.

Conclusion: Processed meat and fish products differ greatly in sodium content across different countries and across different food subcategories. This indicates great

For peer review only

potential for food producers to reformulate the products in sodium content, as well as for consumers to select less salted food.

Keywords: sodium, sodium reduction, processed foods, food reformulation, Food Switch

Strengths and limitations of this study

Strengths :

1) This is the first cross-sectional study to compare the sodium content of processed meat and fish products among five countries.

Potential limitations:

1) Products were obtained only in selected stores at a specific time point in each country.

2) We did not capture food-purchasing data to quantify actual sodium consumption of processed meat and fish products.

3) The data collection time of different countries is inconsistent. During this period, due to the growing interest in reducing salt policies on a global scale, product reformulation may have changed.

Introduction

High sodium intake is the major cause of high blood pressure and increases the risk of cardiovascular disease, renal disease and premature mortality.[1] Processed meat and fish products constitute important categories of processed food, which provide high-quality protein, minerals and vitamins to daily diet. The processing itself offers an opportunity to add savory flavour to food, and prolong the shelf-life of food products to improve food safety. However, the high sodium content, which is known

For peer review only

to be a key factor for quality and sensory attributes of processed meat and fish, otherwise raises a huge public health concern. The global average sodium intake was about 4000mg/d in 2010, twice the maximum 2000mg/d recommended by the World Health Organization (WHO).[2] A previous study conducted in 2013 in China reported that the average sodium content of processed meat and fish products was 1029mg/100g and 1424mg/100g respectively, above half of the recommended daily sodium intake.[3] In developing countries like China, sodium intake mainly derives from cooking, yet with the rapid urbanization and dietary transition, the consumption of hidden sodium in processed foods including meat and fish products tends to be increasing rapidly.[4] In developed countries, where more than three quarters of sodium intake comes from processed foods, it was estimated that sodium intake from meat and meat products contributed approximately 16-25% of total daily sodium intake.[5] In response to the WHO goal of 30% sodium reduction by 2025, various sodium reduction actions have been taken worldwide. It is worth paying attention to the high sodium content of processed meat and fish products.[6]

Many countries have made efforts to reduce the sodium content of processed foods. The United Kingdom (UK), the United States (US) and Australia have set voluntary targets for sodium reduction in various categories of processed foods.[7-9] South Africa was the first to include the statutory maximum sodium targets in several processed food categories.[10] This targets-based approach has been shown to be effective in reducing sodium content for many food products.[11 12] Within the same food category, the sodium level is much lower in food products in countries with sodium reduction targets than those without the target,[4] which can be demonstrated by one in UK vs China: the median sodium content was on average 4.4-fold less in UK sauces compared with their Chinese equivalents.[13]

For peer review only

The George Institute for Global Health established a global food composition database in 2010 as part of The International Network for Food and Obesity/non-communicable diseases Research, Monitoring and Action Support (INFORMAS), with an aim to collate and track the nutritional compositions of processed foods worldwide.[14]The global food composition database uses a standardized methodology for data collection and processing, with data available from more than ten countries as of 2020.[15-17] This makes the comparison of sodium content across countries possible. The five countries cover three developed and two developing countries which allow the comparison meaningful to instruct sodium reduction among countries especially for developing countries. In addition, the selected countries have their own sodium reduction strategies. The comparison results may provide meaningful implication for sodium reduction through pre-packaged food in other countries.

In this study, levels of salt content of processed meat and fish products are compared among five INFORMAS member countries: UK, US, Australia, China and South Africa. These five countries have different sodium reduction strategies and relatively large dataset available for sodium content comparison for processed meat and fish products, which allows for the comparison conductible and meaningful. The purpose of this study is to compare the sodium content level and achievements in sodium reduction for meat and fish products among the five countries, and indicate possible strategies on sodium reduction for different countries.

Materials and Methods

Data Collection

Images of pre-packaged foods were taken using smartphone applications (The George Institute Data Collector and FoodSwitch)[15] by trained data collectors as well as consumers through crowdsourcing and uploaded to a central content management

For peer review only

system. The information displayed on the packages, including product, nutrition and ingredient information, was then entered into a uniform web-based data management system by professionally trained clerks. All entered information was reviewed by a second data entry clerk for accuracy. Products with verified information were classified according to a standard food categorization system. This study used data of processed meat and fish products collected in the UK, Australia, South Africa and China available within the George Institute global food composition database, with the data collection time ranging from 2012 to 2018. We also obtained data on processed meat and fish products from the US through Label Insight Inc. for non-profit research.

Data Categorisation

In the food categorization system, processed meat products and processed fish products fall into two independent categories. Processed meat products were further classified to 16 subcategories: meat alternative products, bacon, canned meat, frozen meat, meat burgers, salami and cured meats, sausage and hot dogs, sliced meat, dried meat, pate and meat spreads, kebabs, other meat products, raw flavoured meats, whole hams and similar products, roasted chicken, and raw unflavoured meats. Processed fish products were divided into 4 subcategories: canned fish, chilled fish, frozen fish and other fish.

Data Exclusion Criteria

Products with no declaration of neither sodium nor salt values were excluded. In the case of identical products with the same sodium content, but available in different package sizes, these were regarded as duplicates and only one product was included.

Data analysis

For peer review only

Sodium value data were obtained from the Nutrition Information Panel (NIP). For products with only salt values available, sodium values were calculated from salt values divided by 2.5. Median and interquartile range (IQR) were used to describe the distribution of sodium values (mg/100g) given the non-normal distribution of the data. The Kruskal-Wallis H test was used to compare differences in sodium values of processed meat and fish products across the five countries. If the difference was statistically significant, post-hoc tests were carried out using Bonferonni correction. The subcategory with data records equal to or less than 5 was excluded from the analysis for subcategory comparisons.

In reference to the "Traffic Light" criteria developed by the UK, sodium level was defined as low (< 120mg/100g), medium (120 ≤ sodium ≤ 600mg/100g), and high (> 600mg/100g); and expressed as green, amber and red accordingly to a horizontal bar chart to show the sodium contents visually.[18] The 2017 UK sodium reduction targets were used to assess the percentage of products reaching the targets across the five countries.[19] The maximum sodium targets of each category were selected for ease of comparison, and the average targets were used where maximum targets were not provided. The Chi-square tests were used to compare the proportion of products that meet the 2017 UK sodium reduction targets.

To measure the sodium burden caused by consumption of processed meat and fish products, a sodium intake contribution value was calculated for each category of food products. It was a ratio of daily sodium intake from 100g product against the WHO maximum sodium recommendation (2000 mg/d), assuming the consumption of processed meat and fish food products for a person were 100g per day. For each category of the food products, the contribution value was calculated as median sodium content (mg/100g) / 2000 (mg/d) * 100%, and was highlighted as red, yellow and

For peer review only

green respectively to represent high (>66%), medium (>33%, ≤66%) and low (≤33%) sodium intake contribution.

A two-sided p-value of less than 0.05 was considered significant in the statistical tests. The analyses were conducted using Stata/SE 14.2 and IBM SPSS 21.0.

Patient and Public Involvement

No patient involved.

Results

A total of 33955 processed meat and fish products were collected from the five countries, of which 7455(21.96%) were excluded because of missing sodium data or duplicate products, leaving 26500 (78.04%) products for analysis in this study (Figure 1). The total number of products per country ranged from 885 for the UK to 17098 for the US (Table 1). The number of products per category ranged from 1 in meat alternative products, kebabs and roasted chicken to 2817 in sausages and hot dogs.

For peer review only

Table 1 Sodium Content of Processed Meat and Fish Products across Five Countries (mg/100g)

	USA (n=17098)			SA (n=946)			Australia (n=5673)			UK (n=885)			China (n=1898)			*K-W H TEST*
	n	median	IQR	n	median	IQR	n	median	IQR	n	median	IQR	n	median	IQR	*p*-Value
**Total**	17098	655	353-981	946	571	362-876	5673	489	335-854	885	432	236-786	1898	1050	774-1473	<0.001
**Meat**	12954	768	474-1071	612	754	518-1020	3836	580	376-990	558	590	275-904	1641	1066	800-1450	<0.001
Meat alternative products	372	478	386-607	—	—	—	—	—	—	10	413	236-550	1	1102	1102	0.175
Bacon	707	1667	1050-1857	36	1018	823-1155	289	1150	1020-1700	43	1612	1140-2162	33	805	750-1000	<0.001
Canned meat	462	607	446-964	44	659	517-855	127	717	483-900	8	275	236-826	48	762	688-887	0.018
Frozen meat	1169	532	400-690	123	460	347-577	875	440	347-560	86	275	236-354	33	160	64-713	<0.001
Meat burgers	824	476	305-647	47	638	500-794	162	475	390-584	6	393	315-472	7	612	486-703	<0.001
Salami and cured meats	554	1607	1357-1750	25	1633	1415-1838	265	1410	1200-1600	5	1573	1376-1612	115	1200	949-1532	<0.001
Sausage and hot dogs	2817	829	696-974	133	814	684-935	456	704	565-900	36	550	452-747	269	991	832-1111	<0.001
Sliced meat	1937	875	750-1088	84	900	745-1100	359	989	816-1100	173	668	590-865	23	1132	845-1250	<0.001
Dried meat	1383	1536	1036-1929	43	2144	1682-2280	126	1760	1400-2000	—	—	—	281	1509	1153-1760	<0.001
Pate and meat spreads	83	679	518-911	14	789	438-861	89	480	310-603	28	629	550-708	5	1916	1670-2490	<0.001
Kebabs	2	493	462-525	—	—	—	38	408	294-504	1	393	393	—	—	—	—
Other meat products	427	589	94-1071	26	865	560-1070	83	570	340-925	13	275	236-472	649	1050	782-1390	<0.001
Raw flavoured meats	501	446	254-750	24	465	356-580	678	368	245-502	27	197	197-315	42	563	382-763	<0.001
Whole hams and similar products	4	848	750-1518	2	839	744-934	80	1080	1000-1400	12	1081	983-1356	103	1039	940-1250	0.977
Roasted chicken	2	563	357-768	1	415	415	37	359	271-548	3	197	118-236	9	893	693-996	<0.001
Raw unflavoured meats	1710	71	63-402	10	69	58-120	172	66	50-323	62	79	79	23	122	70-234	0.011
**Fish**	4144	364	208-529	334	356	265-453	1837	395	286-540	327	354	236-550	257	942	470-1867	<0.001
Canned fish	1219	388	299-467	168	353	280-400	821	380	309-472	66	354	315-393	138	902	599-1586	<0.001
Chilled fish	147	389	171-691	36	449	226-798	332	587	324-917	126	511	157-747	53	1744	370-5072	<0.001
Frozen fish	2733	347	152-541	100	295	169-434	559	340	225-449	117	275	197-354	36	131	73-715	<0.001
Other fish	45	5389	3813-6000	30	451	362-580	125	860	426-4990	18	550	432-747	30	1305	1147-1644	<0.001

For peer review only

Levels of Sodium Content for Processed Meat and Fish across the Five Countries

Table1 shows the sodium content of processed meat and fish products across the five countries. Overall, for processed meat and fish products combined, China had the highest sodium level (1050mg/100g, IQR: 774-1473), ranking as the country with the saltiest products for both meat (1066mg/100g, IQR: 800-1450) and fish products (942mg/100g, IQR: 470-1867), followed by the US, South Africa, Australia, and the UK (432mg/100g, IQR: 236-786). Taking meat products alone, Australia had lower median sodium content (580mg/100g, IQR: 376-990) than the UK (590mg/100g, IQR: 275-904). Significant differences in sodium levels were seen in 18 subcategories among five countries. For example, the sodium content of roasted chicken in China was 4.5 times that of the UK (893mg/100g vs 197mg/100g) ($p<0.001$); chilled fish in China, 4.5 times that of the US (1744mg/100g vs 389mg/100g)($p<0.001$); pate and meat spreads in China, about 4 times that of Australia (1916mg/100g vs 480mg/100g)($p<0.001$). However, the sodium content of bacon, frozen meat, salami and cured meats, dried meat and frozen fish in China was the lowest among five countries. (Figure 2-1, 2-2, 2-3, 2-4, 2-5) Taking bacon as an example, the median sodium contents ordered from highest to lowest were the US (1667mg/100g), the UK (1612mg/100g), Australia (1150mg/100g), South Africa (1018mg/100g) and China (805mg/100g). Within each country, the sodium content also varied greatly across different subgroups with raw unflavoured meats being the lowest sodium content subcategory.

Comparison of Sodium Content Using Traffic Light Criteria

Across the five countries, a large proportion of processed meat and fish products fell into the red and amber categories, with the highest proportion of green light products found in the UK, accounting for 12.66% of all meat and fish products. China

For peer review only

had the largest proportion of red light (85.83%) and the smallest proportion of green light products (3.64%) ($p<0.001$). A similar difference was seen in processed meat products ($p<0.001$). For processed fish products, the highest proportion of green light products was observed in the US (14.50%), followed by 12.84% in the UK. South Africa had the largest proportion of amber light products (84.73%) and the lowest proportion of red light (8.68%) and green light (6.59%) products among five countries ($p<0.001$). (Figure 3-1,3-2,3-3)

Comparison of Sodium Content to 2017 UK Sodium Reduction Targets

In the 13 categories of processed meat and fish products, the countries with sodium contents reaching 2017 UK sodium reduction targets in descending order were the UK (26.6%), Australia (23.2%), South Africa (22.4%), the US (18.4%) and China (7.1%). Statistically significant differences were observed among countries ($p<0.001$) for bacon, canned meat, frozen meat, meat burgers, sausage and hot dogs, other meat products and canned fish. The UK had the highest percentage of products achieving the targets except for bacon products, in which only 14.0% of bacon products in the UK reached the target, lower than that in the US (28.2%), Australia (50.2%), SA (75.0%) and China (84.9%). (Table 2)

For peer review only

Table 2 Number and percentage of products with sodium content meeting the 2017 UK Sodium Targets

Product categories	2017 UK Sodium Targets (mg/100g) [a]	China n (%)	UK n (%)	Australia n (%)	SA n (%)	US n (%)	*p*-Value
Total	—	101(7.1)	135(26.6)	833(23.2)	154(22.4)	1927(18.4)	<0.001
Meat alternative products	500	0(0.0)	5(50.0)	—	—	212(57.0)	0.907
Bacon [b]	1152	28(84.9)	6(14.0)	145(50.2)	27(75.0)	199(28.2)	<0.001
Canned meat	272	0(0.0)	4(50.0)	5(3.9)	0(0.0)	24(5.2)	<0.001
Frozen meat [b]	272	17(51.5)	36(41.9)	87(9.9)	18(14.6)	139(11.9)	<0.001
Meat burgers	352	1(14.3)	2(33.3)	27(16.7)	4(8.5)	249(30.2)	<0.001
Salami and cured meats [b]	652	5(4.4)	0(0.0)	2(0.8)	0(0.0)	18(3.3)	0.08
Sausage and hot dogs	600	7(2.6)	21(58.3)	147(32.2)	14(10.5)	350(12.4)	<0.001
Sliced meat	272	1(4.4)	5(2.9)	9(2.5)	0(0.0)	32(1.7)	0.218
Kebabs	352	—	0(0.0)	14(36.8)	—	0(0.0)	—
Other meat products	300	29(4.5)	8(61.5)	17(20.5)	2(7.7)	150(35.1)	<0.001
Whole hams and similar products [b]	652	0(0.0)	1(8.3)	3(3.8)	0(0.0)	0(0.0)	0.036
Roasted chicken	272	0(0.0)	3(100.0)	10(27.0)	0(0.0)	0(0.0)	0.189
Canned fish [b]	360	13(9.4)	44(66.7)	367(44.7)	89(53.0)	554(45.5)	<0.001

[a] The maximum sodium targets of each category were selected for ease of comparison, and the average targets were used where maximum target was not provided.
[b] Average sodium targets.

Contribution of Sodium Content Per 100g to WHO Daily Sodium Intake Recommendation

Table 3 shows the sodium intake contribution from the consumption of processed meat and fish products. If 100g meat and fish products was consumed, the sodium intake would account for 47.2% of the WHO recommended maximum daily intake (2000 mg/d) on average in China, followed by the US (47.1%), South Africa (36.9%), Australia (34.6%) and the UK (27.1%). Each country had its own major sodium contributors. For example, the sodium contribution values were the highest for pate and meat spreads (95.8%) and chilled fish (87.2%) in China, but very low in the other four countries. Several food categories had relative high sodium intake contribution, highlighted with red or yellow across the five countries. They were dried meat, salami and cured meats, bacon, sliced meat, and whole hams and similar products.

For peer review only

Table 3 Sodium intake contribution values (%) of processed meat and fish products [a]

Food categories	China	UK	Australia	SA	US
All categories	47.2	27.1	34.6	36.9	47.1
Meat alternative products	55.1	20.7	—	—	23.9
Bacon	40.3	80.6	57.5	50.9	83.4
Canned meat	38.1	13.8	35.9	33.0	30.4
Frozen meat	8.0	13.8	22.0	23.0	26.6
Meat burgers	30.6	19.7	23.8	31.9	23.8
Salami and cured meats	60.0	78.7	70.5	81.7	80.4
Sausage and hot dogs	49.6	27.5	35.2	40.7	41.5
Sliced meat	56.6	33.4	49.5	45.0	43.8
Dried meat	75.5	—	88.0	107.2	76.8
Pate and meat spreads	95.8	31.5	24.0	39.5	34.0
Kebabs	—	19.7	20.4	—	24.7
Other meat products	52.5	13.8	28.5	43.3	29.5
Raw flavoured meats	28.2	9.9	18.4	23.3	22.3
Whole hams and similar products	52.0	54.1	54.0	42.0	42.4
Roasted chicken	44.7	9.9	18.0	20.8	28.2
Raw unflavoured meats	6.1	4.0	3.3	3.5	3.6
Canned fish	45.1	17.7	19.0	17.7	19.4
Chilled fish	87.2	25.6	29.4	22.5	19.5
Frozen fish	6.6	13.8	17.0	14.8	17.4
Other fish	65.3	27.5	43.0	22.6	269.5

[a] The contribution value, calculated as median sodium content (mg/100g) / 2000 (mg/d) * 100%, was a ratio of daily sodium intake from 100g product against the WHO maximum sodium recommendation (2000 mg/d), assuming the daily consumption of processed meat and fish food for a person were 100 g per day. The contribution values were highlighted as red, yellow and green to represent high (>66%), medium (>33%, ≤66%) and low (≤ 33%) contribution to sodium intake, respectively.

Discussion

This study provides the first detailed comparison of sodium content in processed meat and fish products among five countries. The results shows extremely wide discrepancy within and between countries. Overall, processed meat and fish products in the UK had the lowest median sodium content, and China had the highest sodium in both meat and fish products. The sodium content of meat and fish products in each country was high compared with "Traffic Light" criteria with only 10% of the products in the UK and US and no more than 5% in China and South Africa falling

For peer review only

into the green light group. The percentage of products meeting 2017 UK sodium reduction targets were generally low ranging from 7.1% (China) to 26.6% (the UK). A 100 g serving size of processed meat and fish products could on average contribute to one half/third of WHO daily maximum sodium intakes in all countries.

The amount of sodium intake from pre-packaged food differs in different countries. In developed countries like Australia, US and the UK, processed foods provide 75%~80% of sodium intake.[20 21] It was reported that processed meat products accounted for about 20% of daily meat consumption and contributed to around 10% daily sodium intake in Australia.[11] In South Africa, processed meat was also a major sodium source other than bread among processed foods which contributed to about 50% of sodium intake.[21] In China, however, 70%~80% of sodium came from home cooking with a remarkable increase from consumption of processed foods and meals out of home in recent years. Sodium intake from packaged meat and fish products is an emerging concern.[22]

One strategy to reduce sodium intake from packaged products is to encourage consumers to replace high-sodium products with low-sodium products. For example, choosing raw unflavoured meats instead of salami and cured meats would decrease the sodium intake from these foods by ten to twenty fold in all five countries. However, different subcategories of meat and fish products have distinct different organoleptic properties, which coupled with the convenience of pre-prepared products, is the main driver for consumers' choice.[23] Therefore, development of new products with the same or better flavor and less sodium should be encouraged. In addition, Front-of-Pack labelling such as Traffic Light and Health Star Rating labelling as well as consumer awareness campaigns may increase consumer acceptability and demand for healthier products.[21 24]

For peer review only

It is not easy to simply replace or reformulate the high-sodium products which already exists for years. However, the large difference in sodium content of similar products in different countries, and the difference in sodium content among different brands within the same countries, indicate that there is still a lot of room for salt reduction. Product features regarding satisfying flavor, texture, safety and stability have been the key considerations for manufacturers, but attention should also be paid for three situations. First, product formulations might have been lagged behind consumers' requirement for less sodium products. Second, many manufacturers may resist reformulation due to unfounded concern for flavor acceptance and safety.[25]Third, a 10-15% reduction in sodium will go undetected, and the product reformulation could be done step by step.[8 25]

Setting sodium targets for processed foods is an effective way to reduce sodium contents of packaged foods.[4 11 13 24] In the five countries, China had the saltiest meat and fish products among the countries, which is likely due to the lack of sodium targets to limit the sodium added to the products. The remaining four countries have set voluntary or mandatory sodium targets for meat and fish products along with comprehensive sodium reduction policies/programs. The UK has issued four sets of voluntary sodium targets for over 80 categories of processed foods since 2006, and has set up a successful sodium reduction model for other countries through this incremental sodium reduction strategy.[7] Following the UK, the US and Australia set the voluntary sodium targets for various processed foods through the National Salt Reduction Initiative in 2008 in the US and the Food and Health Dialogue in 2010 in Australia, respectively.[20 24] South Africa became the first country to regulate legislated sodium limits for a range of food products in 2012.[12] The results of comparing sodium contents against the latest 2017 UK sodium reduction targets

For peer review only

showed that the UK had the highest proportion of products achieving the targets, followed by Australia, South Africa, US and China. This, to some extent, might be relevant to the implementation of the incremental sodium reduction strategies.

Target implementation is also critical. Our results showed that the proportion of meat and fish products that met the sodium reduction targets was low across all the countries. Even for the best, the UK, the target-achieving rate was only 26.6% for all meat and fish products, which was much lower than the target-achieving rate for noodles (90%) and sauces (70%).[4 13] Some subcategories of meat products such as bacon even had the highest sodium content in the UK among the countries. These suggest robust implementation and monitoring of the voluntary targets are needed. The 2017 UK sodium reduction targets were more rigorous compared with that of other countries. Studies have shown that in Australia, South Africa and the US, about half of meat products met their own national targets.[11 12 20] In summary, the sodium lowering targets provide a level playing field within a country. Many food manufacturers are trying to work towards the targets. This finding also indicates that technical issues should not be a barrier for manufacturers to reformulate their foods.

With development and urbanization, more and more countries have realized the increasing challenge of pre-packaged food to health. Although not surprising to many people, the specific findings in this study could be a good reference in developing specific strategies to promote sodium reduction. To achieve this, several questions could be considered. What the gap and space is for a country in sodium reduction for pre-packaged food? Which products should be targeted on first? Whether and how to adopt the target setting strategy, mandatory or voluntary? And how to overcome the barriers from manufacturers who may be reluctant to reformulate their product by arguing that salt reduction would shorten the shelf life?

For peer review only

The key strength of this study is that it is the first cross-sectional survey of the sodium content of processed meat and fish products in five countries. The standardized methods for data collection and processing, including standardized food categorization, have ensured the comparability of the data. There are several potential limitations of this study. First, products were obtained only in selected stores at a specific time in each country, and the selected stores were major supermarket chains with a large market share but could not represent all stores within the countries. Second, we did not capture food-purchasing data to quantify actual sodium consumption of processed meat and fish products, although the crowdsourcing element of the data collection may somehow reflect what consumers have eaten. Future studies should consider using more reliable product sales data or consumption data to estimate the actual sodium intake from processed meat and fish products in each country. Thirdly, the duration of data collection varies from 2015 to 2018 in China, 2013 to 2017 in the UK, 2014 to 2017 in Australia, 2015 to 2017 in South Africa and 2012 to 2017 in the US. During these periods, although very slow, product reformulation may have occurred due to growing global interest in sodium reduction. To make full use of the data and due to the lack of track records for each product, we did not compare the 5 countries over the same time and were not able to identify and exclude the outdated products during analysis.

Conclusions

The sodium content of meat and fish products in all the selected countries was very high with a 100 g serving size of meat and fish products contributing to one half/third of WHO recommended maximum daily sodium intake. There are large differences in sodium levels of packaged foods among the five countries with different sodium reduction policies. This implies that the target-based strategy is

For peer review only

effective in lowering sodium levels in foods. Therefore, setting feasible or further lower sodium targets is urgent. Regular evaluation is also needed to ensure its robust implementation.

Acknowledgments

We thank Prof Feng J He, Monique Tan, Prof Graham MacGregor from Queen Mary University of London provided data of the UK. Prof Bruce Neal from The George Institute for Global Health permitted and coordinated the data sharing. Blaire Jesseph from Label Insight in the US and Terry Harris from Vitality South Africa collaborated with The George Institute in developing the dataset of the US and South Africa, respectively. Fraser Taylor, Kylie Howes and Lizzy Dunford from The George Institute FoodSwitch team mapped the data and directly provided data of Australia, South Africa and US. Yu Liu from Beihang University provided data of China. Huijun Wang from National Institute for Nutrition and Health at Chinese Center for Disease Control and Prevention provided data of China. We appreciate Jimmy Louie from The University of Hong Kong for his contribution to the revision of the very first draft. And thank National Financial Projects for its financial support.

Contributorship statement

Puhong Zhang, Yuxia Ma, Yuan Li, Chunlei Guo designed the study. Liping Huang, Monique Tan, Feng J He, Terry Harris, Graham MacGregor, Yu Liu and Huijun Wang provided the data. Yuzhu Song, Yishan Wang, Jingmin Ding and Le Dong analyzed the data. Yuzhu Song prepared the first draft of the manuscript. Yuan Li, Chunlei Guo, Puhong Zhang and Yuxia Ma made critical revisions to the manuscript. All authors reviewed and approved the final draft.

Competing interests

For peer review only

All authors have completed the ICMJE uniform disclosure form at http://www.icmje.org/coi_disclosure.pdf and declare: no support from any organization for the submitted work; no financial relationships with any organization that might have an interest in the submitted work in the previous three years, no other relationships or activities that could appear to have influenced the submitted work.

Funding

This research was funded by National Key R&D Program of China, grant number 2016YFC1300200, National Financial Projects (131031107000160004) and the National Institute for Health Research (NIHR, NIHR Global Health Research Unit Action on Salt China at Queen Mary University of London) using Official Development Assistance (ODA) funding (16/136/77). The views expressed in this publication are those of the author(s) and not necessarily those of the funders.

Data sharing statement

Nutrition information of 19601 meat and 6899 fish products was collected using the FoodSwitch mobile application from China, the United Kingdom (UK), Australia, South Africa and the United States (US) from 2012 to 2018.The data of China can be linked yli@georgeinstitute.org.cn and zpuhong@georgeinstitute.org.cn.The data of UK can be linked m.tan@qmul.ac.uk; f.he@qmul.ac.uk; g.macgregor@qmul.ac.uk.The data of Australia can be linked ftaylor@georgeinstitute.org.au.The data of South Africa can be linked TerryH@discovery.co.za.The data of US can be linked bjesseph@labelinsight.com. These data are used with the permission of the authors.

For peer review only

Ethics Statement

This study is based on information about packaged food that has been made public and does not involve any personal information.

For peer review only

References

1. Forouzanfar MH, Alexander L, Anderson HR, et al. Global, regional, and national comparative risk assessment of 79 behavioural, environmental and occupational, and metabolic risks or clusters of risks in 188 countries, 1990–2013: a systematic analysis for the Global Burden of Disease Study 2013. *The Lancet* 2015;386(10010):2287-323. doi: 10.1016/s0140-6736(15)00128-2
2. Powles J, Fahimi S, Micha R, et al. Global, regional and national sodium intakes in 1990 and 2010: a systematic analysis of 24 h urinary sodium excretion and dietary surveys worldwide. *BMJ Open* 2013;3(12)
3. Huang L, Neal B, Dunford E, et al. Completeness of nutrient declarations and the average nutritional composition of pre-packaged foods in Beijing, China. *Preventive medicine reports* 2016;4:397-403.
4. Clare F, Karen C, Michelle C, et al. Know Your Noodles! Assessing Variations in Sodium Content of Instant Noodles across Countries. *Nutrients* 2017;9(6):612.
5. Downs SM, Christoforou A, Snowdon W, et al. Setting targets for salt levels in foods: A five-step approach for low- and middle-income countries. *Food Policy* 2015;55:101-08. doi: 10.1016/j.foodpol.2015.06.003
6. Trevena H, Neal B, Dunford E, et al. An Evaluation of the Effects of the Australian Food and Health Dialogue Targets on the Sodium Content of Bread, Breakfast Cereals and Processed Meats. *Nutrients* 2014;6(9):3802-17. doi: 10.3390/nu6093802
7. He FJ, Brinsden H, Macgregor GA. Salt reduction in the United Kingdom: a successful experiment in public health. *Journal of Human Hypertension* 2014;28(6):345-52.
8. Lindberg R, Nichols T, Yam C. The Healthy Eating Agenda in Australia. Is Salt a Priority for Manufacturers? *Nutrients* 2017;9(8):881-.
9. Reeve B, Magnusson R. Food reformulation and the (neo)-liberal state: new strategies for strengthening voluntary salt reduction programs in the UK and USA. *Public Health* 2015;129(4):1061-73.
10. Webster J, Crickmore C, Charlton K, et al. South Africa's salt reduction strategy: Are we on track, and what lies ahead? *S Afr Med J* 2016;107(1):20-21.
11. Emalie, Sparks, Clare, et al. Sodium Levels of Processed Meat in Australia: Supermarket Survey Data from 2010 to 2017. *Nutrients* 2018
12. Peters S, Dunford E, Ware L, et al. The Sodium Content of Processed Foods in South Africa during the Introduction of Mandatory Sodium Limits. *Nutrients* 2017;9(4) doi: 10.3390/nu9040404
13. Tan M, He FJ, Ding J, et al. Salt content of sauces in the UK and China: cross-sectional surveys. *BMJ Open* 2019;9(9):e025623.
14. Available online:https://www.informas.org/modules/food-composition/(accessed on 17 February 2021)
15. Neal B, Sacks G, Swinburn B, et al. Monitoring the levels of important nutrients in the food supply. *Obesity Reviews* 2013;14:49-58.
16. Dunford E. International collaborative project to compare and track the nutritional composition of fast foods. *BMC Public Health* 2012;12(559):559-59.
17. Jones A, Magnusson R, Swinburn B, et al. Designing a Healthy Food Partnership: lessons from the Australian Food and Health Dialogue. *BMC Public Health* 2016;16(1) doi: 10.1186/s12889-016-3302-8
18. Freire WB, Waters WF, Rivas-Mario G, et al. A qualitative study of consumer perceptions and use of traffic light food labelling in Ecuador. *Public Health Nutrition* 2017;20(5):1-9.
19. Pretorius B, Schönfeldt HC. The contribution of processed pork meat products to total salt intake in the diet. *Food Chemistry* 2018;238:139-45. doi: 10.1016/j.foodchem.2016.11.078
20. Charlton KE, Webster J, Kowal P. To Legislate or Not to Legislate? A Comparison of the UK and South African Approaches to the Development and Implementation of Salt Reduction Programs. *Nutrients* 2014;6(9):3672-95.
21. Shao S, Hua Y, Yang Y, et al. Salt reduction in China: a state-of-the-art review. *Risk Management and Healthcare Policy* 2017;10:17-28.
22. Nam KC, Jo C, Lee M. Meat products and consumption culture in the East. *Meat Science* 2010;86(1):95-102.
23. Eyles H, Webster JL, Jebb SA, et al. Impact of the UK voluntary sodium reduction targets on the sodium content of processed foods from 2006 to 2011: Analysis of household consumer panel

For peer review only

data. *Preventive Medicine* 2013;57(5):555-60.
24. Webster J, Trieu K, Dunford E, et al. Salt reduction in Australia: from advocacy to action. *Cardiovascular diagnosis and therapy* 2015;5(3):207-18.
25. Jaenke R, Barzi F, McMahon E, et al. Consumer acceptance of reformulated food products: A systematic review and meta-analysis of salt-reduced foods. *Crit Rev Food Sci Nutr* 2017; 57(16):3357-3372.

For peer review only

Figure 1 Flow diagram of product selection.

(CN: China; UK: The United Kingdom; AU: Australia; SA: South Africa; US: The United States.)

Figure 2-1 Sodium content of bacon among five countries

Figure legend:

■ Bacon

Figure 2-2 Sodium content of frozen meat among five countries

Figure legend:

■ Frozen meat

Figure 2-3 Sodium content of salami and cured meats among five countries

Figure legend:

■ Salami and cured meats

Figure 2-4 Sodium content of dried meat among five countries

Figure legend:

■ Dried meat

Figure 2-5 Sodium content of frozen fish among five contries

Figure legend:

■ Frozen fish

Figure 3-1 Sodium content Traffic Light on processed meat and fish products among five countries

Figure legend:

■Red (High)

■Amber (Medium)

■ Green (Low)

Figure 3-2 Sodium content Traffic Light on processed meat products among five countries

Figure legend:

■Red (High)

■Amber (Medium)

■ Green (Low)

For peer review only

Figure 3-3 Sodium content Traffic Light on processed fish products among five countries

Figure legend:

■Red (High)

■Amber (Medium)

■ Green (Low)

For peer review only

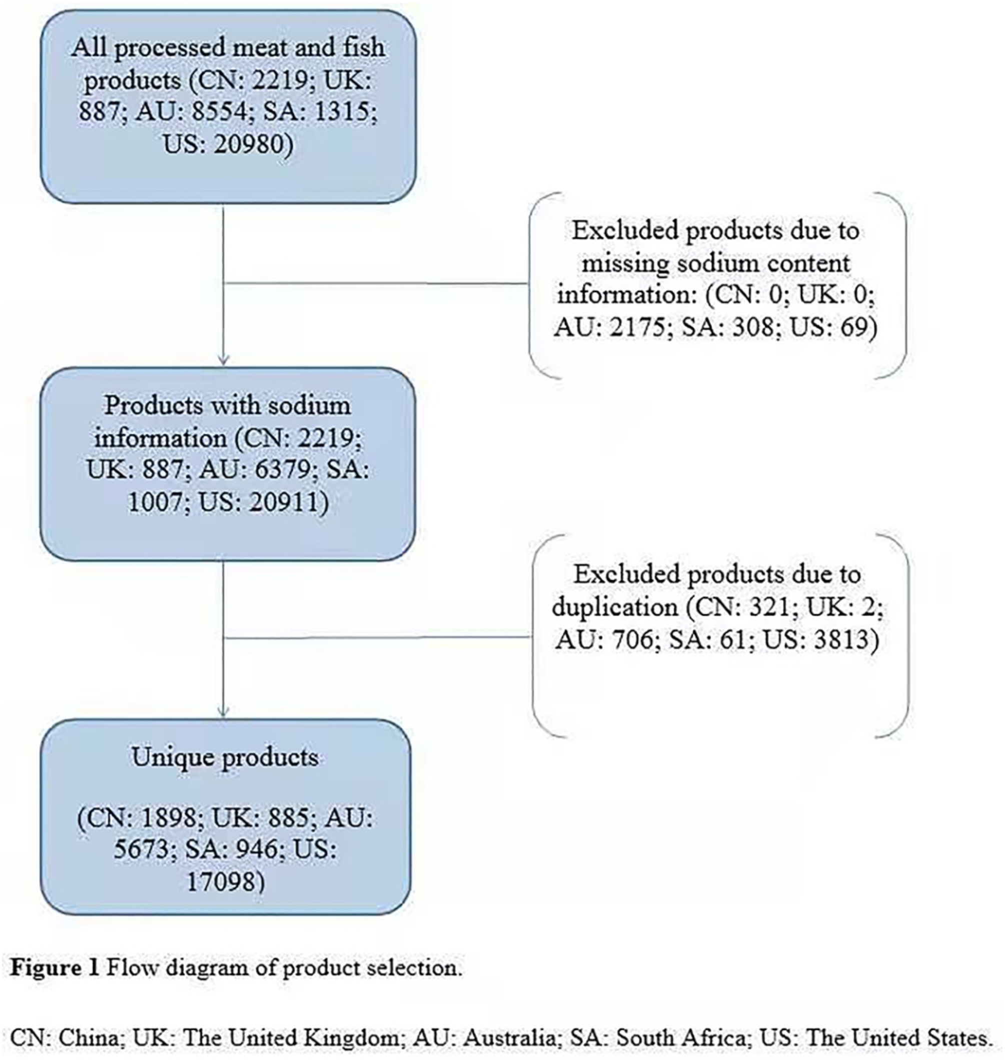

Figure 1 Flow diagram of product selection.
CN: China; UK:The United Kingdom; AU: Australia; SA:South Africa; US: The United States.

101x107mm (300 x 300 DPI)

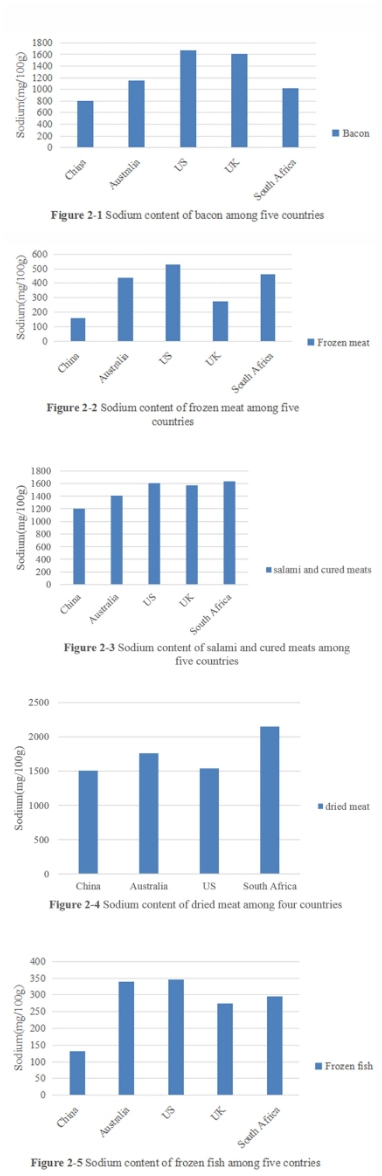

Figure 2-1 Sodium content of bacon among five countries
Figure 2-2 Sodium content of frozen meat among five countries
Figure 2-3 Sodium content of salami and cured meats among five countries
Figure 2-4 Sodium content of dried meat among five countries
Figure 2-5 Sodium content of frozen fish among five countries

152x477mm (300 x 300 DPI)

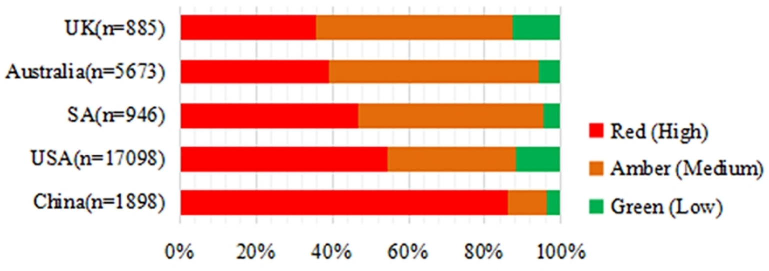

Figure 3-1 Sodium content Traffic Light on processed meat and fish products among five countries

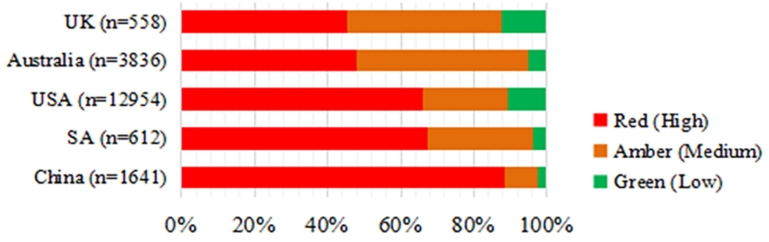

Figure 3-2 Sodium content Traffic Light on processed meat products among five countries

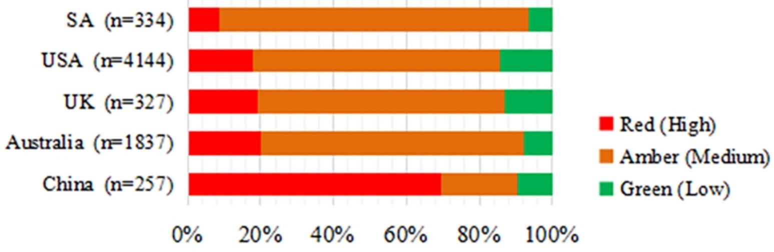

Figure 3-3 Sodium content Traffic Light on processed fish products among five countries

Figure 3-1 Sodium content Traffic Light on processed meat and fish products among five countries
Figure 3-2 Sodium content Traffic Light on processed meat products among five countries
Figure 3-3 Sodium content Traffic Light on processed fish products among five countries

215x320mm (300 x 300 DPI)

STROBE Statement—Checklist of items that should be included in reports of ***cross-sectional studies***

	Item No	Recommendation	Page No
**Title and abstract**	1	(*a*) Indicate the study's design with a commonly used term in the title or the abstract	P1
		(*b*) Provide in the abstract an informative and balanced summary of what was done and what was found	P3-4
**Introduction**			
Background/rationale	2	Explain the scientific background and rationale for the investigation being reported	P4-5
Objectives	3	State specific objectives, including any prespecified hypotheses	P6
**Methods**			
Study design	4	Present key elements of study design early in the paper	P6-7
Setting	5	Describe the setting, locations, and relevant dates, including periods of recruitment, exposure, follow-up, and data collection	P6-7
Participants	6	(*a*) Give the eligibility criteria, and the sources and methods of selection of participants	P7
Variables	7	Clearly define all outcomes, exposures, predictors, potential confounders, and effect modifiers. Give diagnostic criteria, if applicable	NA
Data sources/ measurement	8*	For each variable of interest, give sources of data and details of methods of assessment (measurement). Describe comparability of assessment methods if there is more than one group	P6
Bias	9	Describe any efforts to address potential sources of bias	P5-6
Study size	10	Explain how the study size was arrived at	P6
Quantitative variables	11	Explain how quantitative variables were handled in the analyses. If applicable, describe which groupings were chosen and why	NA
Statistical methods	12	(*a*) Describe all statistical methods, including those used to control for confounding	P7-8
		(*b*) Describe any methods used to examine subgroups and interactions	NA
		(*c*) Explain how missing data were addressed	P7
		(*d*) If applicable, describe analytical methods taking account of sampling strategy	P8
		(*e*) Describe any sensitivity analyses	NA
**Results**			
Participants	13*	(a) Report numbers of individuals at each stage of study—eg numbers potentially eligible, examined for eligibility, confirmed eligible, included in the study, completing follow-up, and analysed	P9
		(b) Give reasons for non-participation at each stage	P9
		(c) Consider use of a flow diagram	P23
Descriptive data	14*	(a) Give characteristics of study participants (eg demographic, clinical, social) and information on exposures and potential confounders	NA
		(b) Indicate number of participants with missing data for each variable of interest	P23
Outcome data	15*	Report numbers of outcome events or summary measures	NA
Main results	16	(*a*) Give unadjusted estimates and, if applicable, confounder-adjusted estimates and their precision (eg, 95% confidence interval). Make clear which confounders were adjusted for and why they were included	P8-17

For peer review only

For peer review only - http://bmjopen.bmj.com/site/about/guidelines.xhtml

		(*b*) Report category boundaries when continuous variables were categorized	NA
		(*c*) If relevant, consider translating estimates of relative risk into absolute risk for a meaningful time period	NA
Other analyses	17	Report other analyses done—eg analyses of subgroups and interactions, and sensitivity analyses	NA
**Discussion**			
Key results	18	Summarise key results with reference to study objectives	P18
Limitations	19	Discuss limitations of the study, taking into account sources of potential bias or imprecision. Discuss both direction and magnitude of any potential bias	P21
Interpretation	20	Give a cautious overall interpretation of results considering objectives, limitations, multiplicity of analyses, results from similar studies, and other relevant evidence	P18-21
Generalisability	21	Discuss the generalisability (external validity) of the study results	P18-21
**Other information**			
Funding	22	Give the source of funding and the role of the funders for the present study and, if applicable, for the original study on which the present article is based	P23

*Give information separately for exposed and unexposed groups.

Note: An Explanation and Elaboration article discusses each checklist item and gives methodological background and published examples of transparent reporting. The STROBE checklist is best used in conjunction with this article (freely available on the Web sites of PLoS Medicine at http://www.plosmedicine.org/, Annals of Internal Medicine at http://www.annals.org/, and Epidemiology at http://www.epidem.com/). Information on the STROBE Initiative is available at www.strobe-statement.org.

For peer review only
